# Evaluation of the Number of Primary Grains in Hypoeutectic Chromium Cast Iron with Different Wall Thickness Using the ProCAST Program

**DOI:** 10.3390/ma16083217

**Published:** 2023-04-19

**Authors:** Edward Guzik, Dariusz Kopyciński, Andriy Burbelko, Andrzej Szczęsny

**Affiliations:** Faculty of Foundry Engineering, AGH University of Science and Technology, al. A. Mickiewicza 30, 30-059 Kraków, Poland

**Keywords:** chromium cast iron, primary austenite grains, chromium carbides, inoculation, EBSD imaging, mathematical structure prediction, cellular automaton (CAFE)

## Abstract

The treatment of inoculation of white cast iron with carbide precipitations that consist of increasing the number of primary austenite grains is not as well-known as the treatment of inoculation of gray cast iron in which the number of eutectic grains increases. In the studies included in the publication, experiments were carried out using the addition of ferrotitanium as an inoculant for chromium cast iron. The Cellular Automaton Finite Elements (CAFE) module of ProCAST software was used in order to analyze the formation of the primary structure of hypoeutectic chromium cast iron in a casting of various thicknesses. The modeling results were verified using Electron Back-Scattered Diffraction (EBSD) imaging. The obtained results confirmed obtaining a variable number of primary austenite grains in the cross-section of the tested casting, which significantly affects the strength properties of the obtained chrome cast iron casting.

## 1. Introduction

The microstructure of white high-chromium cast iron largely depends on the chemical composition of the liquid phase and the direction of change in the chemical composition of the liquid during the crystallization process. The data contained in [1] show that the structure of high-chromium cast iron may have the following phase composition:

1. dendrites α + peritectic (γ—M_7_C_3_) + eutectic (α—M_7_C_3_) + eutectic (γ—M_7_C_3_);
2. primary carbides M_7_C_3_ + peritectic (γ—M_3_C) + eutectic: (γ—M_3_C) + eutectic (γ—M_7_C_3_);
3. primary carbides M_7_C_3_ + peritectic (γ—M_7_C_3_) + eutectic (γ—M_7_C_3_);
4. dendrites γ eutectic (γ—M_7_C_3_);
5. dendrites γ peritectic (γ—M_7_C_3_) + eutectic (γ—M_7_C_3_) + eutectic (γ—M_3_C);
6. primary carbides M_3_C + eutectic (γ—M_3_C);
7. dendrites α + peritectic phase γ peritectic (γ—M_7_C_3_) + eutectic (γ—M_7_C_3_);
8. dendrites γ + eutectic (γ—M_3_C);
9. primary carbides M_7_C_3_ + eutectic (γ—M_7_C_3_);
10. M_7_C_3_ primary carbides + M_3_C primary carbides + eutectic (γ—M_3_C);
11. eutectic (γ—M_7_C_3_).

The described phase composition of chromium cast iron results from the theoretical analysis of cross-sections of the polythermal triple phase equilibrium system of Fe-C-Cr alloys determined for the designed chemical composition. The eutectic structure of white high-chromium cast iron is related to the crystallization of the M_7_C_3_ complex carbide [2].

As a pre-eutectic phase and as a phase included in the eutectic grain, there is M_7_C_3_ carbide, which crystallizes in the form of rods with a hexagonal cross-section. Undoubtedly, the leading phase during the crystallization of the eutectic α + M_7_C_3_ is carbide and it should be expected that eutectic grains will nucleate and grow on its surface. In addition, high-chromium cast iron is a material sensitive to the cooling rate, as well as other types of cast iron. Thus, the morphology of the eutectic crystallization and the phase composition of the microstructure in high-chromium cast iron also depend on the crystallization and cooling conditions of the casting. When a high-chromium cast iron casting is provided with a high cooling rate, the share of the eutectic type γ(α) + (Fe,Cr)_7_C will increase, while the share of the eutectic type γ(α) + (Cr,Fe)_3_C will decrease. Therefore, the microstructure of the casting walls of various dimensions will differ not only in the size of a given phase but also in the phase composition. In general, the structure of white chromium cast iron can be classified according to the range of alloy chemistry, phase growth rate and temperature gradient. These parameters allow hypoeutectic, eutectic and hypereutectic structures to be obtained in cast iron.

Table 1 presents the chemical composition and hardness of abrasion-resistant high-chromium cast iron according to the PN-EN 12513 standard (https://www.en-standard.eu/din-en-12513-founding-abrasion-resistant-cast-irons/ accessed on 5 March 2023 or https://sklep.pkn.pl/pn-en-12513-2011e.html accessed on 5 March 2023). Based on the data presented in Table 1, white high-chromium cast iron covers four ranges of chromium content: the first is 11 wt% < Cr ≤ 14 wt%; the second is 14 wt% < Cr ≤ 18 wt%; the third is 18 wt% < Cr ≤ 23 wt%; and the fourth is 23 wt% < Cr ≤ 28 wt%. However, for each range of chromium content, this standard provides three ranges of carbon content. It should be remembered that as the carbon content decreases, both the plastic properties and the resistance to repeated impact loads increase. However, when the carbon content increases, the hardness of this cast iron also increases. Wear-resistant high-chromium cast iron, on the other hand, is particularly sensitive to the cooling rate. Therefore, the microstructure of high-chromium cast iron is particularly dependent on the thickness of the casting wall, i.e., on the casting modulus, similarly to nickel-chromium (martensitic) cast iron.

To obtain the required hardness given in Table 1, it becomes necessary to optimize the chemical composition, that is, the appropriate selection of alloying elements depending on the thickness of the casting wall. For castings supplied in the unfinished state of high chrome cast iron with a low content of alloying elements, may be difficult to obtain the hardness required by the standard. A similar situation should be expected in the case of castings of this type of cast iron with a high wall thickness. The requirements for such castings should be separately agreed upon between the ordering party and the manufacturer. In addition, it should be noted that the shrinkage of white chrome cast iron of all types during crystallization is significant (about 2%), similarly to the shrinkage of cast steel which requires the use of risers of appropriate dimensions. Castings are usually left in the molds until they reach an ambient temperature. However, it is also allowed to remove castings from the mold at a sufficiently high temperature for the purpose of heat treatment. The addition of additives (recommended by the PN-EN-12513 standard), such as molybdenum and vanadium, to the white high-chromium cast iron can lead to the formation of additional carbides in the discussed structure, or if such carbides do not precipitate, to replace some of the chromium in the M_7_C_3_ type carbides, thereby increasing its share in the metal matrix. Molybdenum is introduced into high-chromium cast iron to prevent the separation of pearlite in the metal matrix of high-chromium cast iron. If pearlite appears in the metal matrix of this cast iron, it will mean a significant decrease in the abrasion resistance of the cast iron.

In the group of high-chromium cast iron resistant to abrasive wear, there is a widespread type 15-3 chromium-molybdenum cast iron (known as Climax Alloy) containing 14 ÷ 16 wt% chromium and 2.4 ÷ 3 wt% molybdenum. Grades of this type of cast iron are included in the US standard ASTM A532 as Class II Types B and D and designated as 15% Cr-Mo-LC (low carbon) and 15% Cr-Mo-HC (high carbon). In fact, it is a grade of white martensitic cast iron and its equivalent, according to the PN-EN 12513 standard, is a grade classified in the group of high-chromium white cast iron as EN-GJN-HV600(XCr14) with some modifications in the composition of alloying elements such as wt% Ni + wt% Mo + wt% Cu. It must be noted that this cast iron shows very good resistance to abrasive wear while maintaining good plastic properties and good corrosion resistance. The molybdenum in this alloy is bound in the form of carbides of the Mo_2_C and (Cr, Fe, Mo)_7_C_3_ types and is partially dissolved in a solid solution. Type 15-3 cast iron can be used in the unfinished state when the wall thickness of the casting is small, with the possible use of stress relief annealing at a temperature of 200–250 °C. To obtain a high hardness value, this type of cast iron is hardened in air and then possibly tempered. In general, it must be said that the PN-EN 12513 standard for high-chromium cast iron castings allows the following types of heat treatment: tempering, hardening, hardening and tempering, soft annealing, soft annealing and hardening, soft annealing, hardening and tempering. However, in the case of the need for mechanical treatment of castings made of high chromium cast iron, soft annealing is recommended. If the order concerns castings after soft annealing, it is the client who is responsible for the correct course of the subsequent treatments: hardening and tempering.

Chromium cast iron is used in the production of castings that must be characterized by very good wear resistance and good corrosion resistance.

The casts produced may be several millimeters thick, for example, the cast wall of a defibrating machine used for the production of wood pulp for the production of MDF plates, which can be up to several dozen millimeters, or the cast of slurry pump bodies used in mines. In articles [3,4], the authors presented the results of research related to the inoculation of cast iron. The study was concerned with the effect of changing the content of the FeTi inoculant on the primary structure of hypoeutectic chromium cast iron. These studies show that this inoculant affects the primary structure of cast iron, that is, the faster nucleation of the primary austenite grains results in an increase in their number. This leads to a reduction in the hot cracking of cast components and reduces the level of porosity in the casting. These unfavorable properties can be eliminated by FeTi inoculation as a result of fragmentation of the primary structure of the casting [3,4]. According to the authors of the article, this is how the effect of using the inoculation procedure on the structure and properties of such alloys should be understood.

The phase composition of chromium cast iron described above results from the theoretical analysis of cross-sections of the polythermal triple phase equilibrium system of Fe-C-Cr alloys determined for the designed chemical composition.

In the literature, another important approach to the inoculation procedure can be found, which consists of the production of other types of carbides in the structure of chrome cast iron, for example, TiC, NbC and others [5,6,7,8,9,10,11,12,13,14,15,16,17,18]. Determining the number of primary phase in hypoeutectic cast iron with austenite-carbide eutectic is problematic. In the case of graphite eutectic, there are simple methods based on counting nodular graphite precipitates (ductile iron) or areas limited by phosphorus eutectic precipitates (flake graphite cast iron). In the case of carbide eutectics, the above-mentioned methods cannot be used. A separate issue is the measurement number and size of primary austenite grains. In order to perform reliable metallographic tests, the use of the Electron Back-Scattered Diagram (EBSD) method is proposed [3]. This method requires the use of a scanning microscope with an appropriate detector and very careful sample preparation. These factors make the analysis of alloys with high abrasion resistance difficult and time-consuming. The solution may be the use of metal crystallization simulation software such as ProCAST by the ESI Group. With the selection of appropriate parameters, the structure [19,20,21,22,23,24,25,26,27] can be simulated, which will be formed throughout the casting volume, allowing the testing time by reducing the number of samples tested.

## 2. Methodology

### 2.1. Simulation Domain

**Figure 1:**
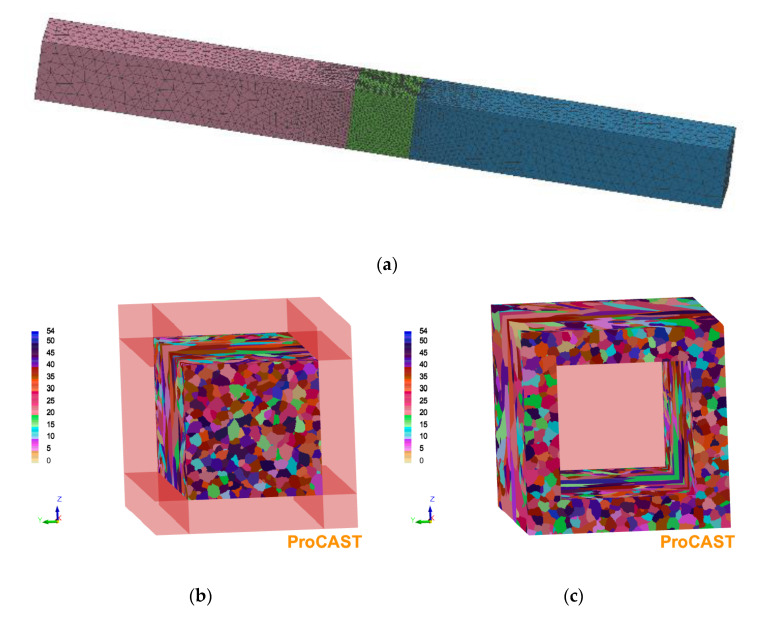
(**a**) An example of a model of a 20 mm thick plate (green) between two layers of 50 mm thick bentonite molding composition; (**b**) typical microstructure in the analyzed area with cross-section 10 × 10 mm; (**c**) 2.5 mm thick outer buffer layer.

In the Laboratory of Computer Modelling of Crystallization Processes, modeling of the primary structure was carried out for the thickness of 20 mm, 30 mm and 40 mm. The model was carried out for a fragment of an infinite plate with the thickness mentioned above, located between the two layers of bentonite mass, 50 mm thick each, as in Figure 1a.

The length of the sample in the direction of heat dissipation (L) is 20, 30 and 40 mm, respectively. The cross-section of the area for which the results of the microstructure simulation with the CAFE method were presented has dimensions of 10 × 10 mm (Figure 1b). In the direction perpendicular to the discharge direction, the heated sample is surrounded on all sides by a 2.5 mm thick buffer layer (Figure 1c), in which the CAFE simulation was also carried out. The total area in which nucleation and grain growth are simulated using the CAFE method is 15 × 15 × L mm.

Few examples are known of the application of the CAFE method to simulate the microstructure of hypoeutectic alloys [28,29,30]. The CAFE module of the ProCAST 2022.0/Visual-Environmemt 18.0 software is designed to predict the structure formed during primary single-phase solidification and does not provide for the possibility of considering eutectic solidification. Due to the relatively small fraction of carbide eutectics, the growth of eutectic colonies in the analyzed alloy begins at the final stage of crystallization, and eutectic structure components are mostly localized at the grain boundaries of the primary solid solution grains. Therefore, in this study, it was decided to apply the above software to evaluate the sizes, distribution and shape of austenite primary grains formed in the alloyed iron castings studied.

The model does not take into account the possibility of nucleation and grain growth processes in the area beyond the outer boundaries of the buffer layers. For this reason, the microstructure of the outer zone of the buffer zone obtained in the simulation is not a good representation of the microstructure of the primary grains. The purpose of using such a buffer area was to eliminate the impact on the microstructure of the analyzed area of the inability to account for the influence of nucleation and grain growth in the area located beyond the outer lateral surface of these buffer areas. When analyzing the simulation results, the microstructure of the buffer zone was not taken into account. All simulation results shown below were obtained in the analysis area with a 10 × 10 mm cross-section.

The sample is located between the two layers of molding sand of the same cross-section and 50 mm thickness in the direction of heat dissipation on each side. The adiabatic condition was assumed on the side surfaces of the entire temperature field modeling area. On the front walls of the molding sand area (15 × 15 mm), the condition of air cooling was adopted.

### 2.2. Café Simulation

For the prediction of the primary grains structure, the Cellular Automata technique is used in the ProCAST software. The processes of the nucleation and growth of the solid phase grains from the liquid are analyzed based on a simulated temperature field. Detailed information on the simulation method using the cellular automaton technique can be found in the publications [31,32,33,34,35].

For the prediction of the temperature field, the Fourier equation is solved numerically by using the Finite Elements Method as follows:

(1) $$\rho_{c}\frac{dT}{d\tau }=div\left(\lambda \cdot gradT\right)+q$$

where:

ρ—density, kg/m^3^; *c*—heat capacity, J/(kg × K);
*T*—temperature, K; τ—time, s;
λ—thermal conductivity coefficient, W/(m × K);
*q*—rate of heat of crystallization release (source function), W/m^3^;
div and grad—mathematical operators of divergence and gradient.

In the model, 3D approach is used for the estimation of the primary austenite grain number and shape with an account of the local undercooling at the solid-liquid interface Δ*T*. The model assumes that the dependence of the fraction of substrates for the heterogeneous nucleation of primary austenite grains on the undercooling at which their activation and nucleation occur follows the Gaussian statistical law. The parameters of this normal distribution are the mean value of undercooling (Δ*T_m_*), at which the rate of increase in the number of growing grains with increasing undercooling increases more rapidly, and the standard deviation (Δ*T*_σ_).

With undercooling increasing, the number of activated nucleation substrates *n,* on which the primary austenite grains nucleate and grow, increases until maximum undercooling is reached before recalescence. The rate of nucleation with increasing undercooling is calculated based on the probability density function of the normal distribution as follows:

(2) $$\frac{dn}{d(∆T)}=\frac{n_{max}}{∆T_{\sigma }\sqrt{2\pi }}\cdot exp\left[-{\left(\frac{∆T-∆T_{m}}{\sqrt{2}∆T_{\sigma }}\right)}^{2}\right]$$

where *n*_max_ is the maximum number of heterogeneous nucleation substrates generated in the alloy as a result of the inoculation treatment.

The number of grains is calculated by integrating Equation (2) during the period of increasing undercooling to a maximum value. Increasing the temperature during recalescence, which causes a decrease in undercooling, terminates the nucleation.

The velocity of the interface migration at the dendrite grain tip (v) is calculated by the Kurz–Giovanola–Trivedi relation [36] as follows:

(3) $$v\left(∆T\right)=a_{2}\cdot ∆T^{2}+a_{3}\cdot ∆T^{3}$$

where *a*_2_ and *a*_3_ are growth kinetics coefficients.

The parameters of the nucleation equations that describe the kinetics and grain growth used in the simulation are summarized in Table 2.

The parameter *n*_max_, which determines the maximum number of substrates for heterogenic nucleation in alloys with technical purity levels, depends on the method of smelting and the subsequent ladle metallurgy processing of the liquid alloy. The goal of inoculation processing is to create more such substrates by introducing master alloys containing substances that form such substrates in greater quantities into the melt. Details of the applied treatment analyzed in this text will be presented below.

To evaluate the effect of the inoculation treatment on the number and size of primary austenite grains in the simulation, three different values of the *n*_max_ parameter were used.

The thermal solution using Finite Elements (FE) and a Cellular Automata (CA) technique for primary microstructure prediction is coupled by the source function term of Equation (1).

The initial alloy temperature was assumed to be 1400 °C, while the initial molding sand temperature was 20 °C. The density of the analyzed alloy used to simulate the temperature field by means of Equation (1) in the temperature range of 1200 to 1400 °C varies from 7180 kg/m^3^ to 6840 kg/m^3^. The corresponding changes in the thermal conductivity coefficient in the same range of temperature changes were 23,000 to 25,100 W/(m·K), and the changes in specific heat were 905 J/(kg·K) to 810 J/(kg·K).

### 2.3. Experimental Verification of Simulation Results

**Figure 2:**
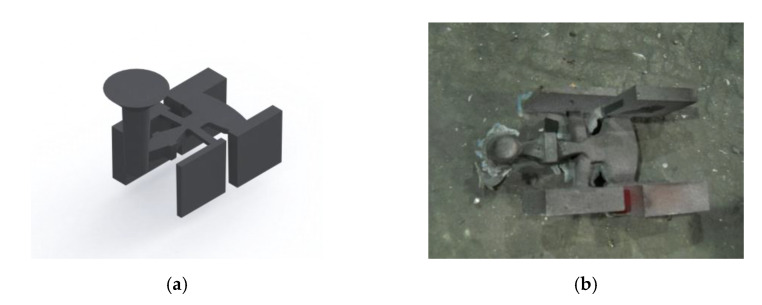
(**a**) Model of the research casting; (**b**) the appearance of the casting immediately after removing the mold.

To verify the results of the simulation, chromium cast iron was melted. Melts were carried out at Odlewnie Polskie S.A. in Starachowice. A medium-frequency induction furnace with a capacity of 120 kg located in the Research and Development Centre of Foundry Components “OBRKO” was used for the tests. Plates with dimensions of 100 × 100 mm and thicknesses of 10, 20, 30 and 40 mm were cast. The appearance of the research casting is shown in Figure 2.

The EBSD microstructure analysis was performed for three thicknesses: 20, 30 and 40 mm. The chemical composition of the inoculated cast iron is presented in Table 3.

From the cast plates, samples were cut for analysis on a scanning microscope with an EBSD detector as follows:

- from a 40 mm plate, the center of the sample is 0.5 of the thickness of the plate;
- from a 30 mm plate, the center of the sample is 0.18 of the thickness of the plate;
- from a 20 mm plate, the center of the sample is 0.12 of the thickness of the plate.

Primary microstructure analysis was performed using a high-resolution scanning electron microscope FEI Quanta 3D FEGSEM with the EDAX Trident system (EDAX Genesis spectrometer, WDS Genesis LambdaSpec spectrometer and EBSD Genesis TSL back-scatter electron diffraction acquisition system) located in the Institute of Metallurgy and Materials Engineering, Polish Academy of Sciences in Kraków.

## 3. Simulation Results

**Figure 3:**
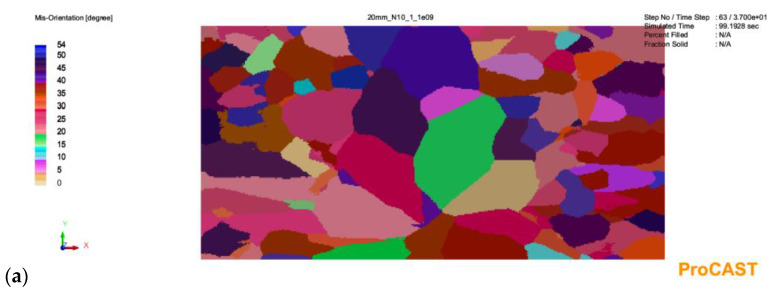

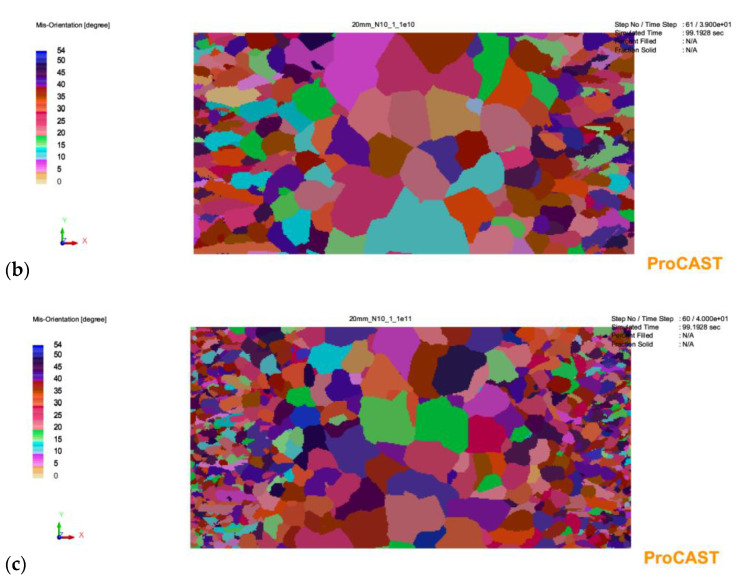
Simulation results for a casting wall thickness of 20 mm with a given number of austenite nucleation sites in the amount of: (**a**)—10^9^; (**b**)—10^10^; (**c**)—10^11^.

**Figure 4:**
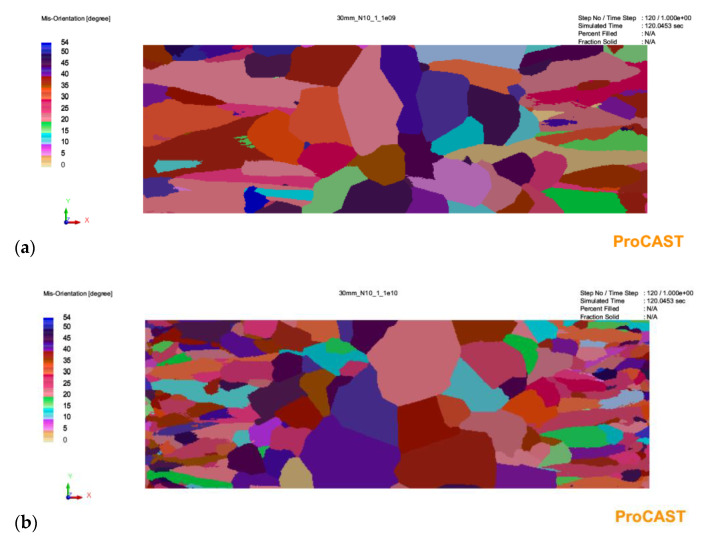

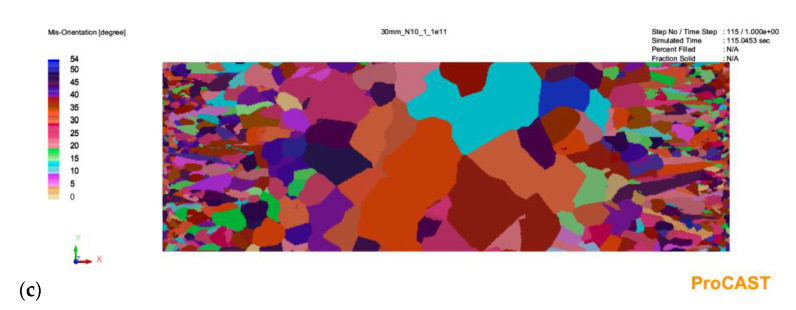
Simulation results for a casting wall thickness of 30 mm with a given number of austenite nucleation sites in the amount of: (**a**)—10^9^; (**b**)—10^10^; (**c**)—10^11^.

**Figure 5:**
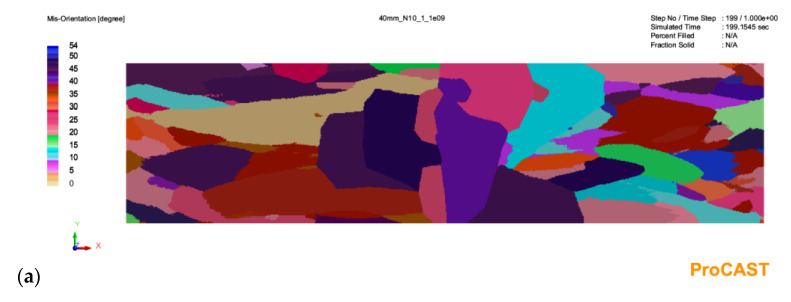

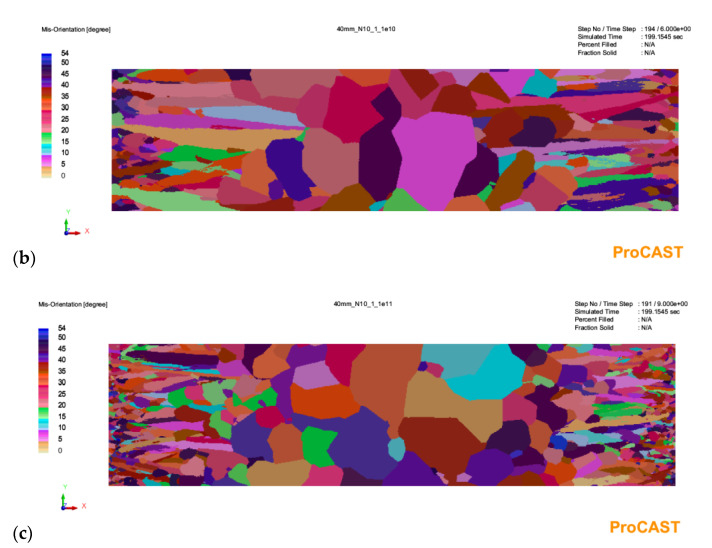
Simulation results for a casting wall thickness of 40 mm with a given number of austenite nucleation sites in the amount of: (**a**)—10^9^; (**b**)—10^10^; (**c**)—10^11^.

The results of the simulation with a given number of nucleation sites from 10^9^ to 10^11^ are shown in Figure 3, Figure 4 and Figure 5.

As can be seen in Figure 3, in the case of a 20 mm thick casting, increasing the number of substrates for heterogeneous nucleation in the analyzed range in the melt volume results in a decrease in grain size. However, it does not change the profile of the microstructure of the primary austenite grains. There are two areas of equiaxial grains and finer grains near the plate axis. For a 30 mm plate casting (Figure 4), increasing the number of substrates not only affects grain size but also causes qualitative changes. For the smallest number (10^9^ m^−3^), there is no zone of equiaxial fine crystals near the casting surface. In this zone, columnar grain structure prevails. The depth of the zone of columnar grains is similar for each analyzed variant. Increasing the number of nucleation substrates results in a marked increase in the number of fine equiaxial grains near the casting surface (Figure 4b,c). The changes in the microstructure of the primary austenite grains for the plate castings look similar: the absence of equiaxial grains near the surface in Figure 5a and an increase in the number of fine equiaxial grains in Figure 5b,c.

## 4. Analysis of the Obtained Microstructures

**Figure 6:**
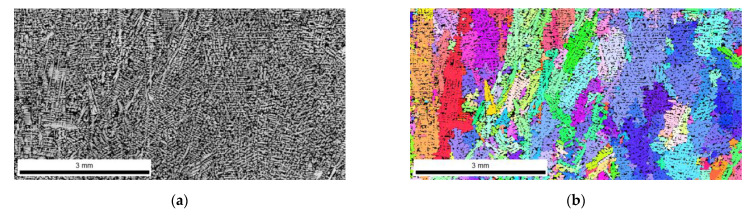
Structure of chromium cast iron in a 20 mm thick plate. (**a**) light microscope; (**b**) EBSD.

**Figure 7:**
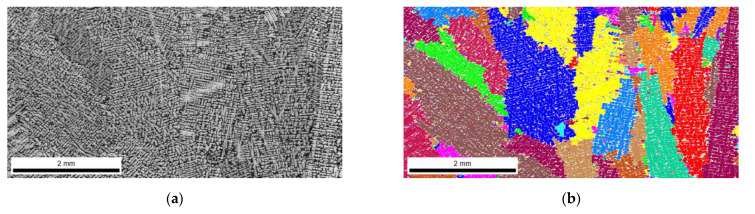
Structure of chromium cast iron in a 30 mm thick plate. (**a**) light microscope; (**b**) EBSD.

**Figure 8:**
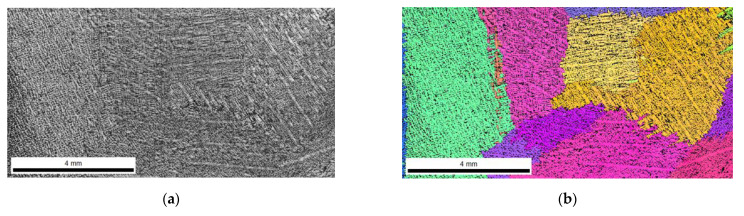
Structure of chromium cast iron in a 40 mm thick plate. (**a**) light microscope; (**b**) EBSD.

**Figure 9:**
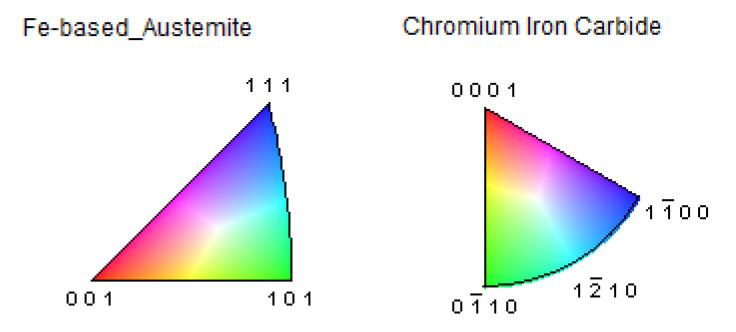
Phase and grain orientation scale.

**Figure 10:**
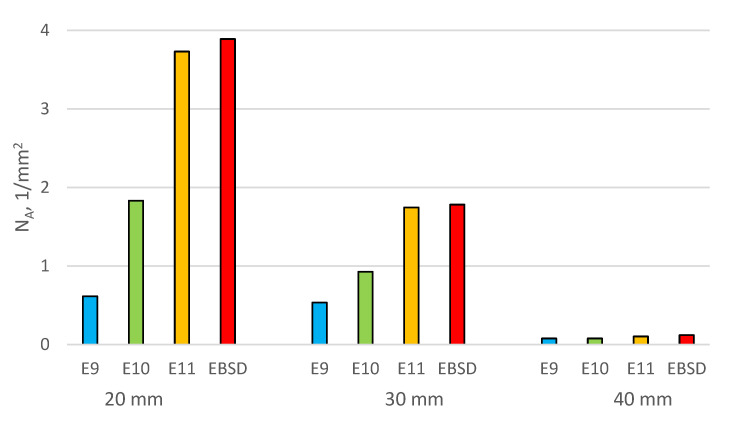
Number of grains of primary austenite N_A_ in the tested samples.

The FEI Quanta 3D FEGSEM high-resolution scanning electron microscope was used for the analysis. Figure 6, Figure 7, Figure 8 and Figure 9 show the microstructure of chromium cast iron using light microscopy and characterize the structure of primary austenite using the EBSD technique in individual plates of variable thickness. The drawings show that the number of primary austenite grains changes with the change in casting wall thickness. The chromium carbide content is low and does not exceed 16% vol. During the calculations, the following assumptions were made so that spatial grain configurations followed the so-called Poisson–Voronoi model [19], as shown in Equations (4)–(6). For each sample, the number of primary austenite grains was calculated, and then the surface grain density N_A_ (5) and the volumetric grain density N_V_ (6) were calculated according to the formula. The calculation results are presented in Table 4 and Figure 10. However, the number of grains in a given area of the metallographic microsection N was calculated according to Formula (4) as follows:

(4) $$N=N_{W}+0.5N_{P}+0.25N_{R}$$

(5) $$N_{A}=\frac{N}{P},\;\frac{1}{{mm}^{2}}$$

(6) $$N_{V}=0.568\cdot {\left(N_{A}\right)}^{\frac{3}{2}},\frac{1}{{mm}^{2}}$$

where:

N—number of grains in the area; N_W_—number of whole grains in the area; N_P_—number of grains cut by sides of the area; N_R_—number of grains in corners of the area; N_A_—surface grain density 1/mm^2^; P—area mm^2^; N_V_—volumetric grain density 1/mm^3^.

**Table 4:** Data and results for calculating numbers of N_A_ and N_V_ grains.

Sample		N	P	N_A_	N_A_	N_V_
			mm^2^	1/mm^2^	1/cm^2^	1/cm^3^
20 mm	E9	26	43.3063	0.6145	61.45	273.65
	E10	77.5		1.8318	183.18	1408.294
	E11	157.88		3.7318	373.18	4094.78
	EBSD	164.6		3.8906	389.06	4358.98
30 mm	E9	7.5	14.033	0.5344	53.44	221.92
	E10	13		0.9263	92.63	506.44
	E11	24.5		1.7458	174.58	1310.29
	EBSD	25		1.7815	178.15	1350.60
40 mm	E9	4.5	58.6762	0.0766	7.66	12.06
	E10	4.5		0.0766	7.66	12.06
	E11	6		0.1022	10.22	18.57
	EBSD	7		0.1192	11.92	23.40

**Figure 11:**
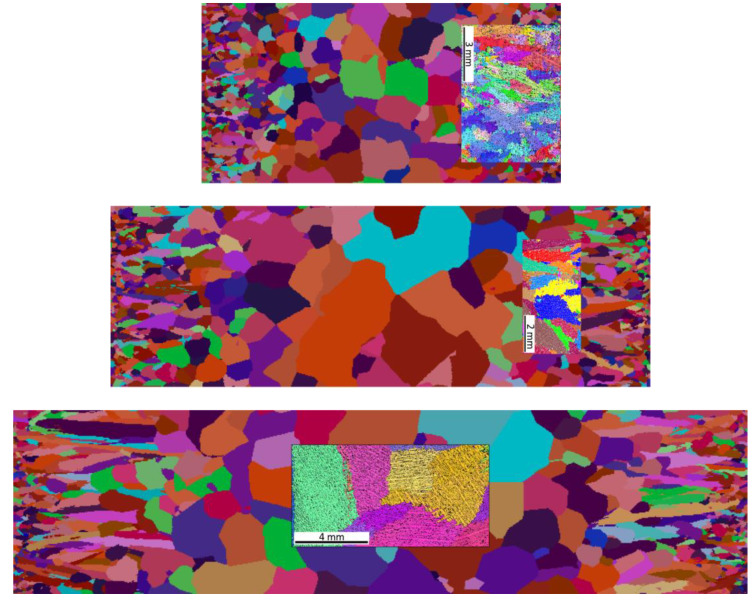
Superimposed images from a cellular automaton simulation with actual EBSD images of the structure.

On the basis of the results, pictures of EBSD microstructures and simulation imaging were matched. The results are shown in Figure 11.

Research has shown that the use of cellular automaton simulation allows analysis of the number of primary austenite grains produced in the structure of a chrome cast iron casting. In addition, the images from the simulation clearly show a large influence of the casting wall thickness on its structure. The number of primary austenite grains at the edge of the wall increases significantly in relation to the central part of the casting. Therefore, the grain measurements made on the central surface of the casting are inadequate for the number of grains located on the edge of the casting.

## 5. Conclusions

1. Simulation with a cellular automaton in the ProCAST program allows for predicting the number of primary austenite grains in chrome cast iron by controlling the number of nucleation sites on the cross-section of the casting.
2. The number of grains determined by the ProCast simulation, after verifying it at selected points of the cross-section of the casting using the EBSD method, allows for faster determination of the number of grains than in the case of using only the EBSD method over the entire cross-sectional area of the casting.

## Figures and Tables

**Table 1:** Chemical composition and hardness of high-chromium cast iron resistant to abrasive wear.

Grade	C	Si	Mn	P	S	Cr	Ni	Mo	Cu
		Max		Max	Max		Max	Max	Max
EN-GJN-HB555 (XCr11)	1.8 ÷ 3.6	1.0	0.5 ÷ 1.5	0.08	0.08	11.0 ÷ 14.0	2.0	3.0	1.2
EN-GJN- HB555 (XCr14)						14.0 ÷ 18.0			
EN-GJN- HB555 (XCr18)						18.0 ÷ 23.0			
EN-GJN- HB555 (XCr23)						23.0 ÷ 28.0			

**Table 2:** Parameters of nucleation and growth kinetics law.

*n*_max_	10^−9^, 10^−10^, 10^−11^	m^−3^
Δ*T_m_*	10	K
Δ*T*_σ_	1	K
*a*_2_	5.042 × 10^−7^	m·s^−1^ × K^−2^

**Table 3:** Chemical composition of hypoeutectic chromium cast iron, wt%.

Inoculant Addition	C	Si	Mn	P	S	Cr	Ti
+0.17% FeTi	1.78	0.79	0.47	0.017	0.01	21.22	0.0599

## Data Availability

Not applicable.
